# Motoneurons innervation determines the distinct gene expressions in multinucleated myofibers

**DOI:** 10.1186/s13578-022-00876-6

**Published:** 2022-08-30

**Authors:** Lei Bai, Wen-Yo Tu, Yatao Xiao, Kejing Zhang, Chengyong Shen

**Affiliations:** 1 Department of Neurobiology in The First Affiliated Hospital; Insitiute of Translational Medicine, Zhejiang University, Zhejiang, China; 2 MOE Frontier Science Center for Brain Research and Brain-Machine Integration, Zhejiang University, Hangzhou, China; 3 Department of Neurobiology, Key Laboratory of Medical Neurobiology of Zhejiang Province, School of Medicine, Zhejiang University, Zhejiang, China

**Keywords:** Neuromuscular junctions, Myonucleus, Denervation, Acetylcholine receptor, Epigenetics

## Abstract

**Background:**

Neuromuscular junctions (NMJs) are peripheral synapses connecting motoneurons and skeletal myofibers. At the postsynaptic side in myofibers, acetylcholine receptor (AChR) proteins are clustered by the neuronal agrin signal. Meanwhile, several nuclei in each myofiber are specially enriched around the NMJ for postsynaptic gene transcription. It remains mysterious that how gene expressions in these synaptic nuclei are systematically regulated, especially by motoneurons.

**Results:**

We found that synaptic nuclei have a distinctive chromatin structure and gene expression profiling. Synaptic nuclei are formed during NMJ development and maintained by motoneuron innervation. Transcriptome analysis revealed that motoneuron innervation determines the distinct expression patterns in the synaptic region and non-synaptic region in each multinucleated myofiber, probably through epigenetic regulation. Myonuclei in synaptic and non-synaptic regions have different responses to denervation. Weighted gene co-expression network analysis revealed that the histone lysine demethylases Kdm1a is a negative regulator of synaptic gene expression. Inhibition of Kdm1a promotes AChR expression but impairs motor functions.

**Conclusion:**

These results demonstrate that motoneurons innervation determines the distinct gene expressions in multinucleated myofibers. Thus, dysregulation of nerve-controlled chromatin structure and muscle gene expression might cause muscle weakness and atrophy in motoneuron degenerative disorders.

**Supplementary Information:**

The online version contains supplementary material available at 10.1186/s13578-022-00876-6.

## Introduction

The neuromuscular junction (NMJ) connects the motoneuron and skeletal muscle, and it controls muscle contraction. NMJ dysfunction causes muscle weakness and is involved in multiple disorders including myasthenia gravis (MG) and amyotrophic lateral sclerosis (ALS) [1, 2]. On skeletal myofibers, AChRs are restricted on the postsynaptic side of NMJs to receive the neurotransmitter acetylcholine from motoneuron axon terminals. Compared with those in the non-synaptic region (NSR), AChRs at the synaptic region (SR) are highly enriched (density: > 10,000 per μm^2^ in SR vs < 10 per μm^2^ in NSR) [3]. One important reason is that motoneurons release agrin and activate its downstream signal to cluster AChR proteins at NMJ sites [1, 4]. Interestingly, in addition to the regulation at the protein level, in situ analysis demonstrates that mRNA of AChR subunits and other NMJ genes (eg. MuSK, Lrp4) are also highly enriched at SR [3, 5, 6], suggesting that there is a special regulation at mRNA levels around NMJs for the synapse assembly and muscle functions.

The myofiber is a long multinucleated cell that results from the fusion of hundreds of mononucleated myocytes. According to different functions and locations in one myofiber, it is composed of the myotendinous junction region, the myofiber body region, and the NMJ region [7]. Total nucleus number of muscle fibers was increased during postnatal development stage [8]. Myonuclei initially align at the center of myofibers after myocyte fusion, but eventually move and anchor to the cell periphery [9]. Interestingly, the distribution of muscle nuclei is not even in a mature myofiber. It has been realized for decades that several nuclei are anchored to stay at the middle of myofibers around NMJs. These nuclei highly express synaptic genes including AChR subunits and thus are defined as synaptic nuclei [3]. In eukaryotic cells, gene expression is closely associated with chromatin structure including euchromatin and heterochromatin, which is highly dynamic responding to different biochemical activities. Electron microscopy analysis found that chromatin compaction is lower in synaptic myonuclei than that in non-synaptic myonuclei [10, 11]. Although it is known that synaptic nuclei are distinct from other nuclei along myotubes [12], it remains mysterious how gene expression in these nuclei is systematically regulated, especially by motoneuron innervation.

Here, we found that synaptic nuclei have a distinct chromatin structure and gene expression profiling compared to those in non-synaptic nuclei. The formation of synaptic nuclei is emerged during NMJ development and maintained by motoneuron innervation. Transcriptome analysis revealed that the number of differentially expressed genes between SR and NSR remarkably reduced upon denervation, probably through epigenetic regulation. Myonuclei in synaptic and non-synaptic regions have different response to denervation. Dysregulation of nerve-controlled chromatin structure and muscle gene expression might cause muscle weakness and atrophy in motoneuron neurodegenerative disorders.

## Materials and methods

### Animals

ChATBCA-eGFP transgenic mice (Jackson Laboratory, stock #007,902) were described previously [13]. SOD1^G93A^ mice were from Shanghai Model Organisms Center. All mice (C57BL/6 J background) were raised under standard conditions with a 12-h light–dark cycle and free accessed to food and water. Both genders were used in the study.

### Isolation of SR and NSR

Mice were sacrificed and the soleus muscles were quickly isolated, followed by stained with R-BTX (1:5000) for 5 min in cold PBS. Two flanks of NMJ region were cut by a scalpel and separated into the BTX-positive region (SR) and the BTX-negative region (NSR) under the stereo fluorescence microscope (SMZ18, Nikon). Samples were stored in liquid nitrogen before RNA extraction or immunoblot.

### Immunofluorescence staining

Whole-mount staining of muscle fibers was performed as described previously [14]. Primary antibodies: anti-neurofilament (1:1000, CST, 2837), anti-SV2 (1:1000, DHSB), anti-H3K9me3 (1:1000, Abcam, ab8898). Secondly antibodies: goat anti-rabbit/mouse IgG conjugated with Alexa Fluor 488 (1:500, Thermo Fisher) or Alexa Fluor 594 (1:500, Thermo Fisher). Other reagents: Rhodamine-conjudated α-Bungarotoxin (R-BTX, 1:2000, Life Sciences); α-Bungarotoxin-ATTO Fluor-488 (BTX-488, 1:2000, Alomone Labs); 4’, 6’-diamidino-2-phenylindole (DAPI, 1 μg/ml in PBS, Sangon Biotech, E607303). Images were obtained with a Nikon confocal microscope (Nikon A1 Ti) and analyzed with the Image J software. The number of synaptic nucleus was counted as previously described. If more than 25% of DAPI-positive area is covered by BTX-positive synaptic site, the nucleus would be considered as a synaptic nucleus [15].

### Western blotting

In brief, acutely isolated soleus muscle was labeled with R-BTX in cold PBS for 5 min, and separated into SR and NSR according to the localization of R-BTX signal under a fluorescence microscope. Tendons at the muscle terminus were excluded. Samples were lysed in the lysis buffer (50 mM Tris–HCl, pH 7.8; 150 mM NaCl; 1% Triton X-100; 0.1% SDS; 1 mM EDTA; 5 mM NaF; 2 mM Na_3_VO_4_; 1 mM PMSF; and protease inhibitor cocktails) and resolved by SDS-PAGE for immunoblot. Primary antibodies: anti-Kdm1a (1:1000, CST, 2184), anti-H3K4me2 (1:1000, Abcam, ab8580), anti-Histone H3 (1:1000, Proteintech, 17,168–1-AP), anti-Neurofilament-L (1:1000, CST, 2837), anti-Myl4 (1:1000, Proteintech, 67,533–1-Ig), anti-α-tubulin (1:5000, Proteintech, 66,031–1-Ig), anti-GAPDH (1:5000, Proteintech, 60,004–1-Ig). Secondary antibodies: HRP-conjugated goat anti-mouse/rabbit IgG (1:5000, Thermo Fisher, 31,430 and 31,460). Immunoreactive bands were visualized by using SuperSignal West Femto maximum sensitivity substrate (34,095, Thermo Fisher). Autoradiographic films were scanned with a Canon MF3010 scanner.

### Sciatic nerve transection

Adult ChATBCA-eGFP mice were denervated as previously described [16]. Mice were anesthetized with 1.5% isoflurane and 2% oxygen using an anesthesia machine (RWD Life Science, R580). The sciatic nerve in the right mid-thigh was exposed and 2-mm sciatic nerve was surgically resected. The proximal end of sciatic nerve was sutured with biceps femoris muscle using 9–0 nylon to ensure complete denervation [17]. The left hind limb from the same mouse was exposed without resection and used as the sham control. Skins were closed with a 4–0 medical silk thread. Mice were recovered from anesthesia on a warm pad before returning to cages. Mice were analyzed at 3, 7, 16, and 30 days after denervation.

### RNA-seq analysis

Adult male ChATBCA-eGFP mice were denervated as above mentioned. After three days, soleus muscles from left (sham) and right (denervated) hind limbs were acutely isolated and separated into SR and NSR in cold PBS. Soleus muscles were chosen because they were easily denervated and their AChRs were convenient to be identified under the fluorescence microscope compared with other skeletal muscles. There were four groups including: (1) innervated synaptic region (SR); (2) innervated non-synaptic region (NSR); (3) denervated synaptic region (DSR); (4) denervated non-synaptic region (DNSR). For each group, soleus muscles from three mice were pooled as one sample. Totally three samples (from nine mice) for each group were individually homogenized in TRIzol reagent (Ambion) and RNA was extracted.

RNA sequencing and analysis were performed by BGI, China. In brief, total RNAs were checked using a NanoDrop and Agilent 2100 bioanalyzer (Thermo Fisher). Isolated RNAs were purified using Oligo(dT)-attached magnetic beads and DNaseI, then the mRNA was fragmented into short fragments as templates for cDNA synthesis. End-repair and 3’ adenylation were applied to these cDNA, followed by ligating bubble adaptors to the ends of these 3’ adenylation cDNA fragments. Subsequently, the double-stranded PCR products were heat-denatured and circularized by the splint oligo sequence. These single-stranded circular DNAs were constructed as the final library for Agilent Technologies 2100 bioanalyzer validation. Ultimately, DNA nanoballs (DNBs) were made and loaded for sequencing and further data analysis on the BGISEQ-500 platform. To determine if the raw data produced by BGISEQ-500 was suitable for subsequent analysis, quality control was performed on the base quality and nucleotide composition of sequencing using software SOAPnuke. After filtering, these clean reads were mapped to the recent Mus musculus mm10 reference genome by Hierarchical Indexing for Spliced Alignment of Transcripts (HISAT) and Boetie2. Gene expression levels were calculated by using the FPKM method (the fragments per kilobase of transcript per million fragments). The differentially expressed genes (DEGs) were identified by DEseq with a cutoff of more than 1.3 fold change and a q-value of less than 0.001. Three biological repeats per group were performed for RNA-seq and analysis. GO analysis were performed using Clusterprofiler package.

### Weighted gene co-expression network analysis (WGCNA)

WGCNA was performed as previously discribed [18, 19]. In brief, FPKM values were log-transformed (logFPKM + 1) before co-expression netwrok construction. The genes were filtered by median absolute deviation (MAD) with the top 11,000 genes being selected for WGCNA using the R package (1.70–3). A soft power threshold of 12, was used to transform the correlation matrix into an unsigned weighted adjacency matrix, leading to a scale-free topology of the network. Subsequently, the dynamic branch cutting with a merging threshold of 0.25 plus a minimum module size of 30 was applied to generate 8 modules.

### STRING

The online STRING 11.5 database (https://string-db.org/) was used to identify chromatin modification. The input options were set to default with a confidence level of 0.4. The network was constructed in Cytoscape v. 3.8.2 using STRING-plugin. The hub genes in the network were identified using maximal clique centrality (MCC) algorithm in cytohubba-plugin.

### Measurement of the ATP levels

ATP levels weremeasured using a bioluminescence detection kit (S0027; Beyotime). The ATP levels of muscle lysate samples were measured in a luciferase reaction based on the production of light caused by the reaction of ATP with added luciferase and D-luciferin. Briefly, samples were incubated with the ectonucleotidase inhibitor ARL 67,165 trisodium salt hydrates (A265; Sigma-Aldrich) to inhibit ATP hydrolysis. ATP was measured by a luciferase reaction in which 560 nm light was emitted when D-luciferin was converted to oxyluciferin. Luminescence was measured using a luminometer (SpectraMax M5/M5e; Molecular Devices). ATP was calculated based on a calibration curve with standard samples. The total amount of proteins was used for normalization.

### Real-time PCR

The synthesis of total cDNA and real-time PCR was performed as described previously [20]. GAPDH was used as the internal control. The primer information is in Table 1.

**Table 1:** Primer information

**Primer**	**Sequence (5'—> 3')**
Chrna1-F	CTCTCGACTGTTCTCCTGCTG
Chrna1-R	GTAGACCCACGGTGACTTGTA
Chrnb1-F	CTCCAACTATGATAGCTCGGTGA
Chrnb1-R	CAGGTCTAAGTACACCTTTGTGC
Chrng-F	CATCTCCTCAGTCGCCATCC
Chrng-R	CACGACCACAGAGTTCACGA
Chrnd-F	GAATGAGGAACAAAGGCTGATCC
Chrnd-R	GGTGAGACTTAGGGCGACAT
Chrne-F	CTATTTCCCCTTTGACTGGC
Chrne-R	CCTCCCTCATAGCGGCGAAT
Tom20-F	GCCCTCTTCATCGGGTACTG
Tom20-R	ACCAAGCTGTATCTCTTCAAGGA
Sdhaf2-F	CATTCAGACGCTTCTACAGAGG
Sdhaf2-R	TCAGGCGATCATAGAGGTTCAG
Npm1-F	ATGGAAGACTCGATGGATATGGA
Npm1-R	ACCGTTCTTAATGACAACTGGTG
Crot-F	AGAACGGACATTTCAGTACCAGG
Crot-R	TTCCAGCCAGTTTCGTTTTCC
Ube2b-F	AAATAAACCACCAACCGTTAGGT
Ube2b-R	TCTCTTCTCATACTCCCGTTTGT
Srf-F	GGCCGCGTGAAGATCAAGAT
Srf-R	CACATGGCCTGTCTCACTGG
Baz2a-F	CCTAACGTGGCCTACGACTG
Baz2a-R	CCCCAAGGATACCGTTGAGC
Ehmt2-F	CCGCCGAGAGAGTTCATAGC
Ehmt2-R	GGTTCGTCCCCGATGAGTG
Id1-F	CCTAGCTGTTCGCTGAAGGC
Id1-R	CTCCGACAGACCAAGTACCAC
Sptbn1-F	GAGTCCAGTCCTGTTCCCTCT
Sptbn1-R	CTTGGCATCTTTATAGAAGCCCA
Dysf-F	AAGACATCAGCCATCGAATTGAG
Dysf-R	CTGCTCCAGGTTAGCTTCCAG
Dync1h1-F	GGGATGAGTATGCCACGCTG
Dync1h1-R	TGTCCTTGAGCCCCTCTGAG
Lrp4-F	GCACACGGAATAGCCAGCA
Lrp4-R	GGATACAGGTACATTCGCCAAG
Musk-F	ATTCTGAGCGTGGAAGACAGT
Musk-R	CAGGGCACCACAACTCTCC
Dok7-F	TGTCTCAGGCTGTTATGCTGG
Dok7-R	CAGTGCGTAGCGGATACGG
Rapsyn-F	GCAGTGCCATGGAGTGTTGT
Rapsyn-R	GGCAAAGCAGAGCAGACAGAGT
Cycs-F	CCAAATCTCCACGGTCTGTTC
Cycs-R	ATCAGGGTATCCTCTCCCCAG
Chchd4-F	GGAAGGGAAGGATCGGATCAT
Chchd4-R	CCCGTGCTCCTCATAGGGA
Chchd10-F	CAGCCGGGTCTTATGGCTC
Chchd10-R	CAGGCTCTGAATTTCCCCCAC
Kdm1a-F	GTGGTGTTATGCTTTGACCGT
Kdm1a-R	GCTGCCAAAAATCCCTTTGAGA
Kdm1b-F	GTACCGGAAATGTGAAAAGGCA
Kdm1b-R	ATACCATCGGGAGGTGTAACC
Kdm2a-F	GAAGAAAGGATTCGGTACAGCC
Kdm2a-R	CCCCGCTGGATATACTCTACA
Kdm2b-F	GATGCTGAGCGGTATCATCCG
Kdm2b-R	GAGACAGCGATCCATGAGCAG
Kdm3a-F	CAGCAACTCCATCTAGCAAGG
Kdm3a-R	TGTTCTCGGTACTTCAGGTTTTG
Kdm3b-F	TCAATATCCACTGGGTCTGTCG
Kdm3b-R	TGCCCCTTTGCACACTTCAG
Kdm4a-F	TAATGTCTGAAAGCGGCTTCTG
Kdm4a-R	TTCTGTTTGGAAACTGACCAGG
Kdm4b-F	AGGGACTTCAACAGATATGTGGC
Kdm4b-R	GATGTCATCATACGTCTGCCG
Kdm4c-F	TGGAGAGTCCCCTAAATCCCA
Kdm4c-R	CCTTGGCAAGACCTGCTCG
Kdm4d-F	CAGAGGCCATCAATTTTGCCA
Kdm4d-R	TTTCCACAGTTCATATCGCTCAG
Kdm5a-F	TGCAAATGAGACAACGGAAAGG
Kdm5a-R	CTGTCATCGCACCCATCACA
Kdm5b-F	CTGGGAAGAGTTCGCGGAC
Kdm5b-R	CGCGGGGTGAAATGAAGTTTAT
Kdm5c-F	TTCAGGTTTACTCCCCGAATCC
Kdm5c-R	CCGCCGTTCTACATTGGGAAT
Kdm5d-F	AGTTGTTCGTACAAACCAGTGT
Kdm5d-R	CTGGCGTCCAACAGGTAGC
Kdm6a-F	AAGGCTGTTCGCTGCTACG
Kdm6a-R	GGATCGACATAAAGCACCTCC
Kdm6b-F	AGTGAGGAAGCCGTATGCTG
Kdm6b-R	AGCCCCATAGTTCCGTTTGTG
Kdm6c-F	GCAGCCTGGATGGACTTAGG
Kdm6c-R	GCTCTGCGGGTATTGGTAGG
Kdm7a-F	ACGACGTGAACCGCTTCAT
Kdm7a-R	GCCGTGTAGAGCTGCACAG
MyHC-IIb-F	TTGAAAAGACGAAGCAGCGAC
MyHC-IIb-R	AGAGAGCGGGACTCCTTCTG
Gapdh-F	ATGGTGAAGGTCGGTGTGAAC
Gapdh-R	AGTGGAGTCATACTGGAACATG

**ChIP Primer**	**Sequence (5'—> 3')**
Chrna1-F	AGGGCAGGATTTGTGATCTG
Chrna1-R	ACGGAAGGCATGCTTCATAC
Chrnb1-F	AAAGGGCCTGAGGAAAAGTG
Chrnb1-R	AGCAGATGTAGGTGGAAACGTC
Chrng-F	TTGATCAGTCACCACCAACC
Chrng-R	AGACTCAGCAAATGGCCAAG
Chrnd-F	AAAGCATACACTGCCACACC
Chrnd-R	TTGAGCCATCTTGCTAGTGC
Chrne-F	AATCCACAAGGCCTGTCATC
Chrne-R	TAACCAATACAGGGCAGCAG

### ORY-1001 treatment

For behavioral and immunostaining analysis, adult wild-type male mice were injected with 20 μl ORY-1001 (MCE, 1.5 μg/μl in 0.5% methyl cellulose) or same amount of 0.5% methyl cellulose at three sites evenly in TA muscles of each hind limb. The control group, n = 11 mice. The ORY-1001-treated group, n = 11 mice. For real-time PCR analysis, denervated TA muscles in the left hind limb of the adult wild-type mouse were injected with ORY-1001 (20 μl, 1.5 μg/μl in 0.5% methyl cellulose). Denervated TA muscles in the right hind-limb from the same mouse was injected with the vehicle control. After three days, TA muscles were harvested for real-time PCR analysis.

### ChIP (Chromatin immunoprecipitation) assay

C2C12 myotubes were treated with ORY-1001 (1.5 μg/μL) for 72 h and cell were lyzed for ChIP assay using the anti-H3K4me2 antibody (1:1000, A2356, ABclonal). The primer information is in Table 1.

### Grip strength measurement

Male mice were subjected to grip strength by a grasping force measuring instrument (47,200, Ugo Basile). When the mice grasp a metal grid that is connected to a force transducer, their tails are gently pulled horizontally to produce a force until the grip is released. Five consecutive trials were performed. ORY-1001-treated mice were tested on Day0 (just before the treatment), Day7 and Day25.

### Rotarod test

Rotarod test was performed as previously described [21]. Male mice were habituated to stay on the spindle for adaptation and training the day before testing. The speed of the rod was 12 rpm per minute. The latency to fall (time) was recorded when the mouse fell off the device. At the start of formal testing, the longest running time was set at 10 min. Five consecutive trials were performed each time. ORY-1001-treated mice were tested on Day0, Day7 and Day25.

### Statistical analysis

Data were analyzed by two-tailed unpaired Student’s *t* test, one-way, or two-way ANOVA. Data were shown as mean ± SEM. Graphpad Prism 6 was used for statistical analysis and statistical significant difference was considered when p < 0.05, unless otherwise indicated. The p values were presented as *p < 0.05, **p < 0.01, ***p < 0.001.

## Result

### Differences in nuclei between synaptic and non-synaptic regions

**Fig. 1:**
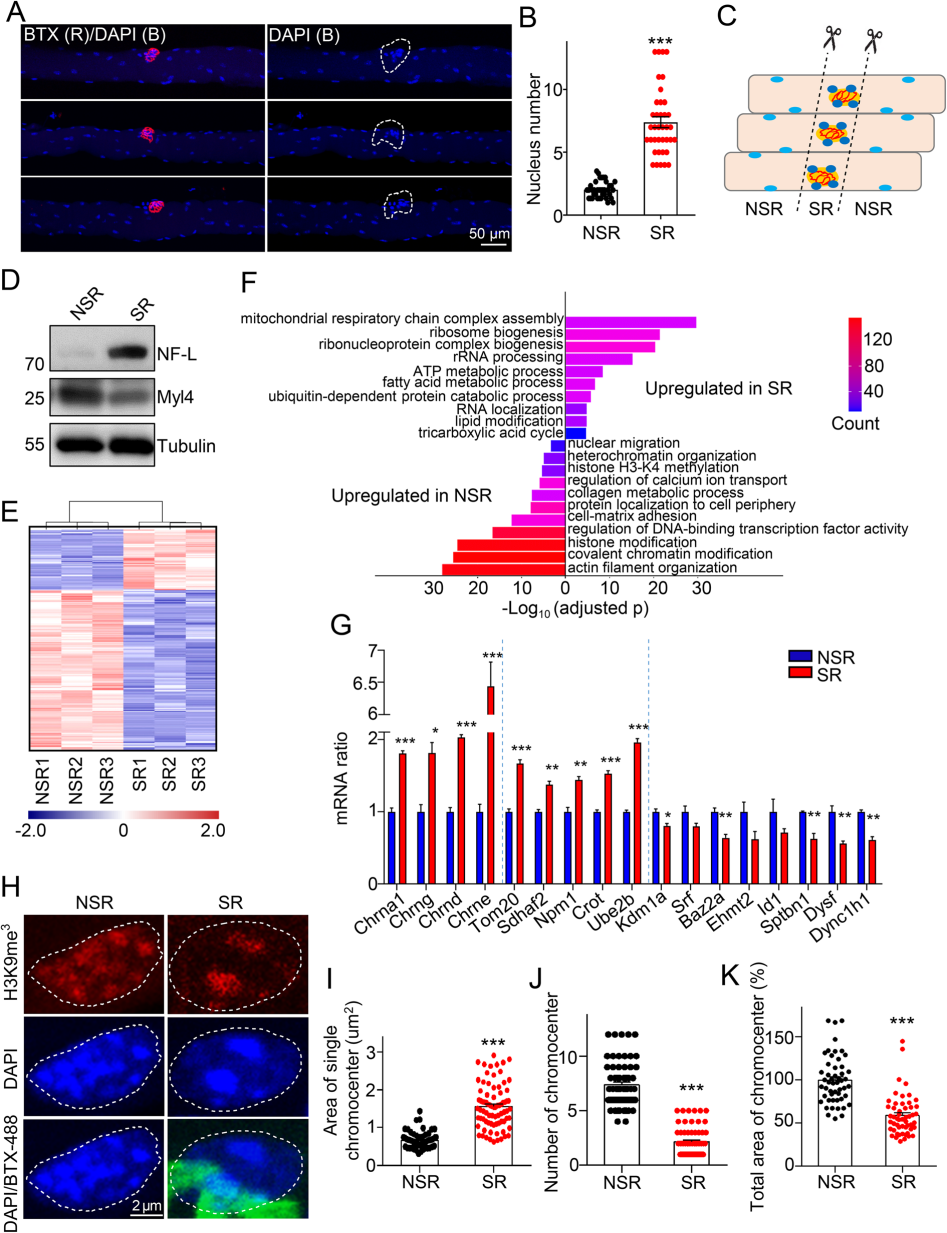
Difference of nucleus between synaptic and non-synaptic regions at NMJs. **A** Whole-mount staining of soleus muscles from 2-month-old C57BL/6 J mice. Note that several nuclei are enriched at R-BTX-labeled synaptic sites (indicated with dotted lines). R-BTX (red). **B** Quantification of number of nucleus in the synaptic region (SR) or the non-synaptic region (NSR) in A. n = 38 myofibers. Results were from three mice. **C** Schematic diagram of the separation of SR and NSR. **D** Immunoblot showing that the expression of NF-L and Myl4 in SR and NSR. Tubulin was used as a loading control. **E** Heat-map of RNA-seq results showing differentially expressed genes (DEGs) in SR and NSR of soleus muscles. Fold change (FC) > 1.3; q < 0.001. Muscle samples (SR and NSR) from nine mice were randomly divided into three groups, and mRNA from each group of samples was individually extracted and processed for RNA-seq. **F** Gene Ontology (GO) analysis showing the enriched biological processes of DEGs between SR and NSR. **G** Verification of DEGs in SR and NSR by real-time PCR analysis. **H** Fluorescence image showing the different chromatin organization in SR and NSR. The heterochromatin is indicated by anti-H3K9me3 (red), which shows a similar pattern of DAPI staining (blue). BTX-488 (green). **I**–**K** Quantification in **H**. (**I**). Area of each chromocenter. n = 76 nuclei in NSR and n = 79 in SR. (**J**). Number of chromocenter. n = 63 nuclei in NSR and n = 65 in SR. (**K**); total area of chromocenter in each nucleus. n = 51 nuclei in NSR and n = 53 in SR. Data were from three mice. Unless otherwise specified, at least three independent experiments were performed; the mean ± SEM is shown; *t-test* in B, G, I, J, and K; *p < 0.05, **p < 0.01, and ***p < 0.001

Different from most types of cells, skeletal myofibers contain multiple nuclei. In soleus muscles and diaphragm muscles from adult mice, nuclei are located peripherally along myotubes (Fig. 1A and Additional file 1: Fig. S1A). NMJ sites in myotubes were indicated by R-BTX, a chemical to specifically label AChR clusters at the postsynapse. Consistent with previous reports [22], several myonuclei were enriched around the NMJ region in the myofiber (Fig. 1A and B, Additional file 1: Fig. S1A and S1B).

To analyze the differences of these nuclei from others in muscles, muscle fibers from soleus muscles were labeled with BTX for 5 min, and SR/NSR was acutely isolated under the fluorescence microscope as indicated (Fig. 1C and Additional file 1: Fig.S1C). Tendons at the muscle terminus were removed. Immunoblot showed that the neuronal protein NF-L (Neurofilament light chain) was mainly expressed in SR, while the non-neuronal protein Myl4 (Myosin light chain 4) was mainly expressed in NSR, indicating a successful separation of samples (Fig. 1D). RNA-seq analysis revealed a distinct difference in the global transcriptomic expression profile between SR and NSR. Totally 1392 genes were up-regulated in SR and 3700 genes were up-regulated in NSR (FC > 1.3, q < 0.001. Figure 1E). GO enrichment analyses were carried out to uncover the functions of these differentially expressed genes (DEGs) (Fig. 1F). Interestingly, up-regulated genes in SR were mainly related to biological processes such as mitochondrial respiratory chain complex assembly, ribosome biogenesis, ATP and fatty acid metabolic process. The result suggests that there is a strong energy demand and active protein synthesis at the synapse. Some genes known to highly express in SR such as Chrnα1, Chrnε and Chrnδ were verified by real-time PCR (Fig. 1G). We also found a higher level of mitochondria protein Tom20 and ATP concentration in SR than those in NSR (Fig. 1G and Additional file 1: Fig. S1D-S1F). This is consistent with our recent findings that enrichment of mitochondrial proteins at NMJs [20]. Up-regulated genes in NSR were mostly related to actin filament organization (Fig. 1F), suggesting the critical role of the cytoskeleton in myotubes for muscle contraction. Interestingly, it was noticeable that chromatin modification, histone modification, and heterochromatin organization were also highly enriched in NSR, suggesting the different chromatin structures between the two regions (Fig. 1F).

DAPI is a fluorescent stain that binds to DNA and shows strong staining with heterochromatin [23], which is indicated with the heterochromatin marker H3K9me3 (Fig. 1H). These DAPI dots are also known as chromocenters, comprising satellite DNA and proteins such as HMGA1, help to contain DNA inside the nucleus between cell divisions [24]. Interestingly, chromocenters in the synaptic nucleus were different from those in NSR (Fig. 1H). Compared with those in NSR, the area of each chromocenter was bigger in SR (0.664 ± 0.022 μm^2^ in NSR vs 1.556 ± 0.066 μm^2^ in SR, Fig. 1H and I), while the number of chromocenters was less in single nucleus (7.365 ± 0.263 vs 2.154 ± 0.161, Fig. 1H and J). The total area of chromocenters in each nucleus in SR was 73.8% of that in NSR (Fig. 1H and K), suggesting less compact chromatin in SR. Taken together, these results demonstrate that myonuclei in SR and NSR have distinct chromatin structures and gene expression patterns.

### Specialization of synaptic nuclei during NMJ development

**Fig. 2:**
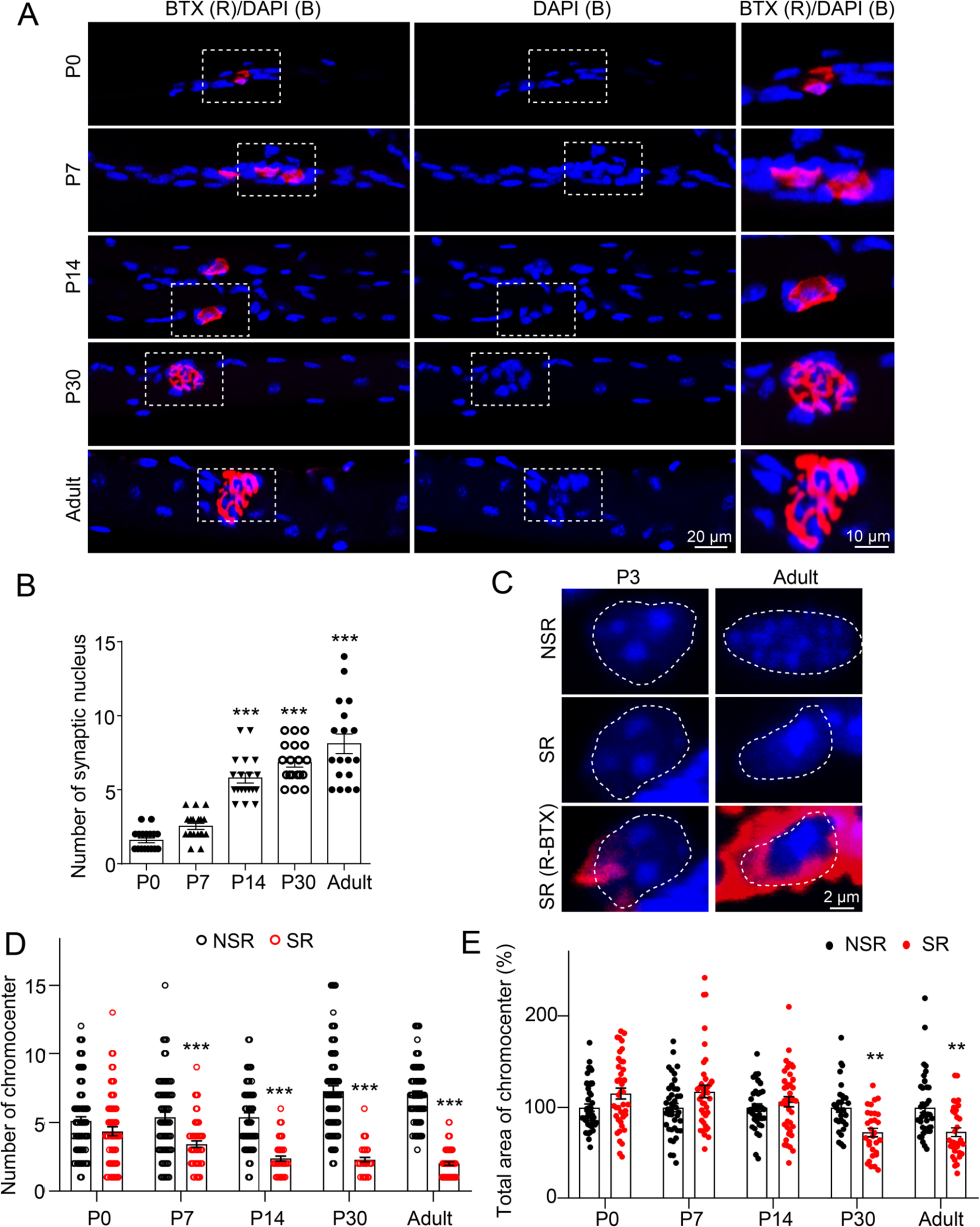
Specialization of synaptic nuclei during development. **A** Whole-mount staining of tibialis anterior (TA) muscles from mice at different developmental stages (P0, P7, P14, P30, or adult). BTX (red), DAPI (blue). The boxes are enlarged. Note that synaptic nuclei were enriched around NMJs in myofibers around P14. P, postnatal. **B** Quantification in A. Images were from three mice. n = 19 NMJs in P0; n = 19 NMJs in P7, n = 19 NMJs in P14, n = 19 NMJs in P30, n = 18 NMJs in adult. One-way ANOVA with Tukey’s post hoc test for multiple comparisons F (4,89) = 57.25. **C** DAPI staining in SR/NSR in P3 and adult TA muscles. DAPI (blue), R-BTX (red). **D** Quantification in A. n = 77 nuclei in NSR from P0, n = 61 nuclei in SR from P0; n = 66 nuclei in NSR from P7, n = 54 nuclei in SR from P7; n = 55 nuclei in NSR from P14, n = 51 nuclei in SR from P14; n = 67 nuclei in NSR from P30, n = 59 nuclei in SR from P30; n = 70 nuclei in NSR from adult, n = 63 nuclei in SR from adult. Images were from three mice. Two-way ANOVA with Sidak’s post hoc test for multiple comparisons: localization F (1,613) = 285.2; developmental time F (4,613) = 2.775. **E** Quantification in A. n = 43 nuclei in NSR from P0, n = 40 nuclei in SR from P0; n = 41 nuclei in NSR from P7, n = 40 nuclei in SR from P7; n = 37 nuclei in NSR from P14, n = 40 nuclei in SR from P14; n = 30 nuclei in NSR from P30, n = 30 nuclei in SR from P30; n = 40 nuclei in NSR from adult, n = 33 nuclei in SR from adult. Images were from three mice. Two-way ANOVA with Sidak’s post hoc test for multiple comparisons: localization **F** (1,364) = 0.8689; developmental time F (4,364) = 8.918. Unless otherwise specified, three independent experiments were performed; the mean ± SEM is shown; *p < 0.05, and ***p < 0.001. ns, non-significant

NMJs undergo an enormous transformation in morphology after birth. We next examined when synaptic nuclei are enriched and specialized during development. Tibialis anterior (TA) muscles from P0, P7, P14, P30, and adult mice were isolated and analyzed. In TA muscles from neonatal mice (P0 and P7), AChR clusters were exhibited as plaques and myonuclei were distributed evenly along myofibers (Fig. 2A and B). In P14 muscles, muscle nuclei started to centralize around AChR clusters. In P30 and adult muscles, AChR clusters were pretzel-like structures, and the enrichment of synaptic nucleus around NMJs became more evident (Fig. 2A and B). Mouse NMJs undergo synapse elimination and tremendous alternation in morphology between one and two weeks after birth, suggesting that muscle nucleus enrichment might be related to the maturation of synapse structure and function. The number and area of chromocenters in synaptic myonuclei and non-synaptic myonuclei in neonatal mice were similar (Fig. 2C–E). However, the number of chromocenters decreased in adult SR (4.344 ± 0.344 in P0 vs 2.000 ± 0.130 in adult, Fig. 2D), while increased in adult NSR (5.117 ± 0.309 in P0 vs 7.029 ± 0.259 in adult, Fig. 2D). The total area of chromocenters in each nucleus in SR was lower than that in NSR in adult (Fig. 2E). These results suggest that chromatin structures in NSR and SR are both dynamically regulated during NMJ maturation.

### Motoneuron innervation is required for the specialization of synaptic nuclei

**Fig. 3:**
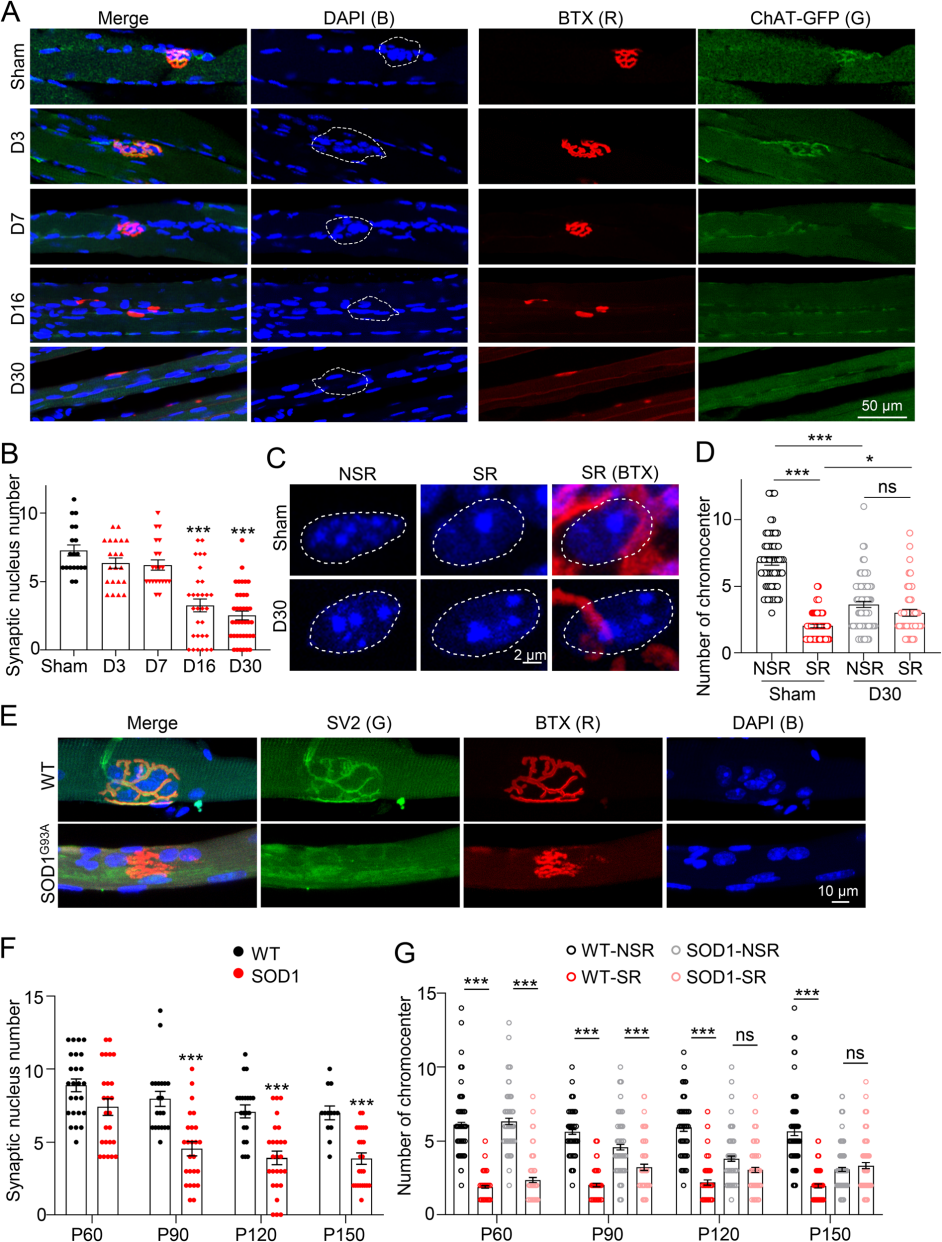
Motoneuron innervation is required for specialization of synaptic nucleus. **A** Whole-mount staining of TA muscles in ChATBCA-eGFP mice (2-month-old) after denervation for 3, 7, 16, or 30 days. BTX (red), DAPI (blue), ChAT-eGFP (green). Note that synaptic nucleus were gradually dispersed. D, denervation. **B** Quantification in A. n = 19 NMJs in Sham, n = 21 NMJs in D3, n = 21 NMJs in D7, n = 30 AChR cluster area in D16, n = 38 AChR cluster area in D30. Images were from three mice. One-way ANOVA with Tukey’s post hoc test for multiple comparisons F (4,127) = 26.46. **C** DAPI staining in SR/NSR in sham and denervated muscles. DAPI (blue), R-BTX (red). **D** Quantification in C. Sham, n = 63 nuclei in NSR and n = 63 nuclei in SR; D30, n = 74 nuclei in NSR and n = 49 nuclei in AChR cluster area. Images were from three mice. Two-way ANOVA with Tukey’s post hoc test for multiple comparisons. SR&NSR: F (1,245) = 183.3; Innervation & Denervation: F (1,245) = 22.01. **E** Whole-mount staining of TA muscles in 4-month-old SOD1^G93A^ mice and littermate controls. BTX (red), DAPI (blue), SV2 (green). SV2 (Synaptic vesicle glycoprotein 2) is a synaptic protein to indicate presynapse at nerve terminals. **F** Quantification of synaptic nucleus number in SOD1^G93A^ mice at indicated ages. Control mice: n = 25 NMJs (or images) in P60, n = 20 in P90, n = 21 in P120, n = 13 in P150. SOD1^G93A^ mice: n = 25 NMJs (or images) in P60, n = 25 in P90, n = 26 in P120, n = 24 in P150. Images were from three littermate mice. Two-way ANOVA with Sidak’s post hoc test for multiple comparisons. Age: F (3,171) = 14.2; genotype: F (1,171) = 64.38. **G** Quantification of synaptic nucleus number in SOD1^G93A^ mice at indicated ages. Control mice: n = 130 nuclei in NSR and 120 in SR in P60, n = 102 nuclei in NSR and 94 in SR in P90, n = 72 nuclei in NSR and 55 in SR in P120, n = 70 nuclei in NSR and 60 in SR in P150. SOD1^G93A^ mice: n = 96 nuclei in NSR and 89 in SR in P60, n = 103 nuclei in NSR and 70 in SR in P90, n = 109 nuclei in NSR and 86 in SR in P120, n = 128 nuclei in NSR and 78 in SR in P150. Images were from three littermate mice. Two-way ANOVA with Tukey’s post hoc test for multiple comparisons. Age: F (3,1446) = 9.626; SR&NSR: F (3,1446) = 313.1. Unless otherwise specified, three independent experiments were performed; the mean ± SEM is shown. ***p < 0.001, ns, no significance

The innervation of motoneurons plays a fundamental role in NMJ formation and maturation [25]. We next asked whether innervation is required for synaptic nucleus enrichment and maintenance of chromatin structure specialization. TA muscles in adult ChATBCA-eGFP mice were denervated for 3, 7, 16, or 30 days (D3, D7, D16, or D30) before analysis. GFP expression in ChATBCA-eGFP mice is driven by ChAT promotor and is restricted in cholinergic neurons in the peripheral nerve system [13]. Consistent with our previous findings [20, 26], GFP-positive motoneurons were degraded in the first week after denervation, while the morphology of AChR clusters was still kept intact (Fig. 3A and S2A). After two weeks of denervation, NMJs became fragmented and eventually dispersed, accompanied with muscle atrophy (Fig. 3A and Additional file 2: Fig. S2A). The average size of one adult NMJ in mice is around 750 µm^2^ [27], thus, we counted the muscle nucleus number in area of 750 µm^2^ in BTX-remaining regions. Strikingly, the number of synaptic nuclei kept intact in D3 and D7 TA muscles, but showed dispersion in D16 and D30 samples (Fig. 3A, B, and Additional file 2: Fig. S2B). Compared with sham controls, the difference of chromocenters in SR and NSR were diminished in D30 myofibers (Fig. 3C and D).

Motoneuron denervation and muscle nucleus defects are often reported in neuromuscular disorders [28]. To examine whether synaptic nuclei are dysregulated in pathological conditions, we checked synaptic nuclei in TA muscles in SOD1^G93A^ mice, an ALS model [29]. Indeed, muscle strength was reduced in SOD1^G93A^ mice (Additional file 2: Fig. S2C). Compared with those in wild-type controls, NMJs lost pretzel-like morphology in denervated myofibers in SOD1^G93A^ mice at the end stage of disease (Fig. 3E and Additional file 2: Fig. S2D). Synaptic nuclei were gradually dispersed during disease progression (Fig. 3F and Additional file 2: Fig. S2E), and the number of chromocenters gradually increased in synaptic myonuclei but reduced in non-synaptic myonuclei (Fig. 3G and Additional file 2: Fig. S2E), similar to those in mice with the surgery of sciatic nerve transection (Fig. 3C and D). Together, our data suggest that motoneuron innervation maintains the enrichment of postsynaptic myonuclei and their unique chromatin structures.

### Innervation determines the gene expression profiles between synaptic and non-synaptic regions

**Fig. 4:**
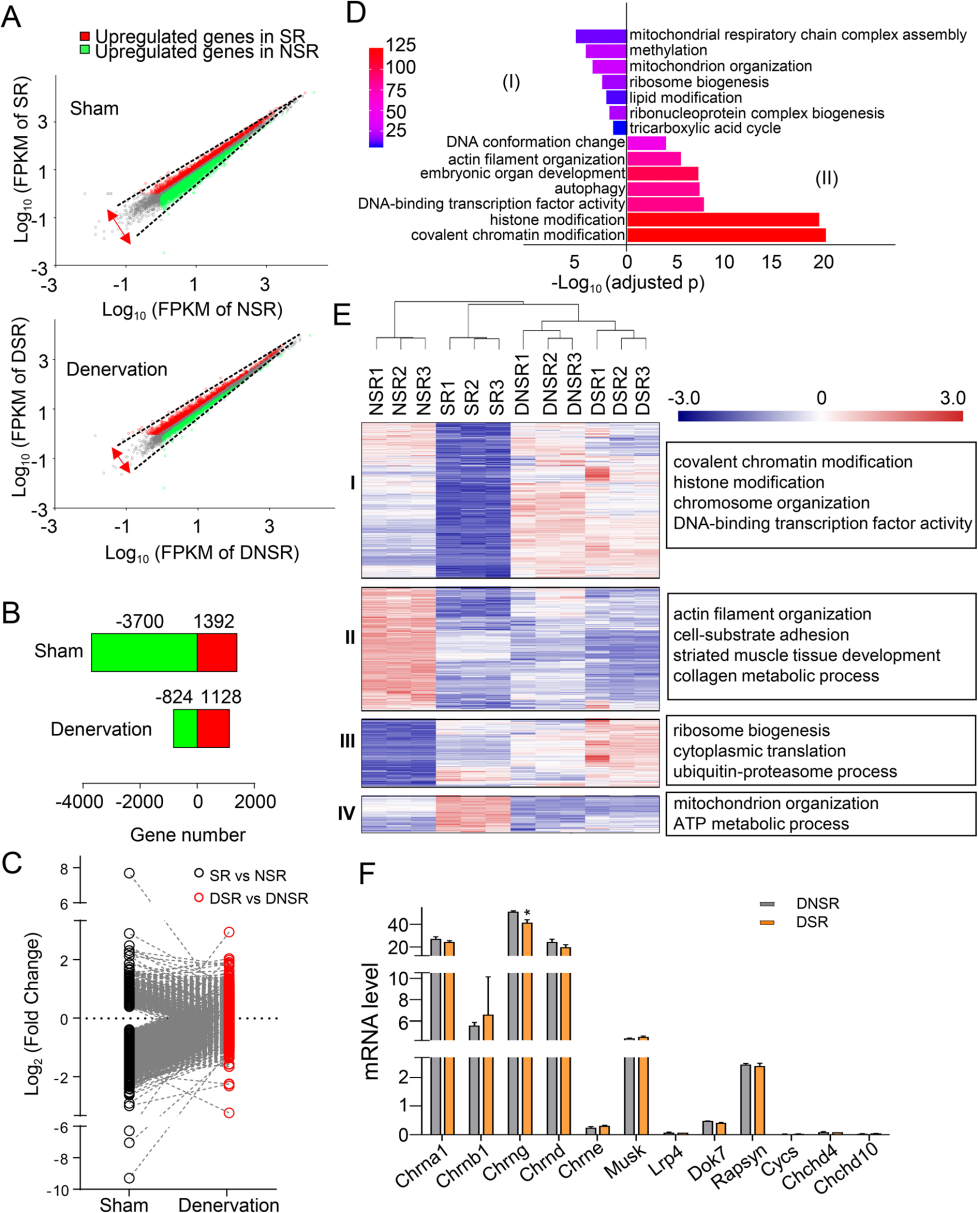
Innervation determines the different expression between synaptic and non-synaptic region. **A** Scatter plots of log 10 (FPKM) obtained from innervated samples (SR and NSR) and denervated samples (DSR and DNSR). Red, up-regulated genes in SR or DSR. Green, up-regulated genes in NSR or DNSR. Note that the scatter plot of DNSR vs DSR samples is narrower than that of NSR vs SR samples, indicating less differences in gene expression between the two regions after denervation. FPKM ≥ 1, FC > 1.3, q < 0.001. **B** Histogram of DEGs number in SR and NSR before and after denervation in A. **C** Scatter plot shows the trend of alteration of DEGs between SR and NSR after denervation. Black, DEGs between SR and NSR in the sham group. Red, corresponding genes in the sham group after denervation. Note that difference in DEGs in the sham group was reduced after denervation. **D** GO analysis of genes that reduced their difference after denervation in **C**. Note that enriched items were opposite in SR and NSR in normal conditions with innervation in Fig. 1F. (I). genes that upregulated in SR (vs. NSR) while lost their difference after denervation. (II). genes that upregulated in NSR (vs. SR) while lost their difference after denervation. **E** Heat-map of RNA-seq of DEGs in SR and NSR in sham or denervated samples. Hierarchical cluster analysis of these DEGs uncovered the unique changes in pathways across four categories (I-IV, listed on the right). **F** Real-time PCR analysis of representative synaptic gene expression in SR and NSR after denervation. Three repeats, mean ± SEM, *t-test*, *p < 0.05

To further clarify how motoneuron regulates early gene expression profiles in SR and NSR, soleus muscles were denervated for 3 days when synaptic nuclei are still enriched at NMJ sites (Fig. 3A). Strikingly, scatter plots showed that the global diversity between SR and NSR were largely diminished after denervation (Fig. 4A). The numbers of DEGs were reduced from 5092 in the innervation group to 1952 in the denervation group. In detail, the number of up-regulated genes in SR decreased from 1392 to 1128 after denervation. The number of up-regulated genes in NSR reduced dramatically from 3700 to 824 genes after denervation (Fig. 4B). Most of the original DEGs (DEG_SR-NSR_) were deprived of their differences and converged in expression after denervation (DEG_DSR-DNSR_). In other words, the difference in expression levels of these DEGs became smaller after denervation (Fig. 4C). We performed GO enrichment analysis of these genes that lost their difference in response to denervation. Intriguingly, most of the items after denervation in SR and NSR showed reverse trend compared to normal conditions with innervation (Figs. 4D and 1F), indicating that the denervation diminishes the distinction between two regions by converting their features.

The expression patterns of all those 5092 DEG_SR-NSR_ after denervation were further exhibited by the heat-map (Fig. 4E). Hierarchical cluster analysis of these DEGs uncovered the unique changes in pathways across four categories (I-IV) (Fig. 4E). For those DEGs that upregulated in NSR during innervation (I and II), they either upregulated together (I) or downregulated together (II) in DSR and DNSR after denervation. For those upregulated genes (I), GO analysis revealed that they were closely related to covalent chromatin modification, histone modification, and chromosome organization, suggesting that the gene expression mediated by innervation may largely depend on epigenetic mechanisms (Fig. 4E). Whereas, for those downregulated genes (II), they were related to actin filament organization, cell-substrate adhesion, striated muscle tissue development, and collagen metabolic process, suggesting that myofibers structure-related genes are inhibited after denervation (Fig. 4E). For those DEGs that upregulated in SR during innervation (III and IV), they also either upregulated together or downregulated together in DSR and DNSR after denervation. For those upregulated genes (III), they were related to ribosome biogenesis and cytoplasmic translation, particularly in DSR, suggesting that SR-enriched proteins are induced by the new synthesis in whole myotubes after denervation, probably as a compensation response of muscles to the denervation (Fig. 4E). The induction of the ubiquitin–proteasome process might explain the muscle atrophy after denervation [30, 31]. Whereas those downregulated genes (IV) were related to mitochondrion organization and ATP metabolic process (Fig. 4E). It suggested a reduction in metabolism after denervation. Notably, reductions in metabolism are also highlighted in muscular atrophy [28], a pathological condition that can also be caused by inactive muscles. Consistent with GO analysis results, real-time PCR also showed that unique expression of NMJ genes in SR was disappeared by either induced or inhibited in two regions of denervated myofibers (Fig. 4F). In summary, our data demonstrate that motoneuron innervation determines the differential gene expression between synaptic and non-synaptic regions.

**Fig. 5:**
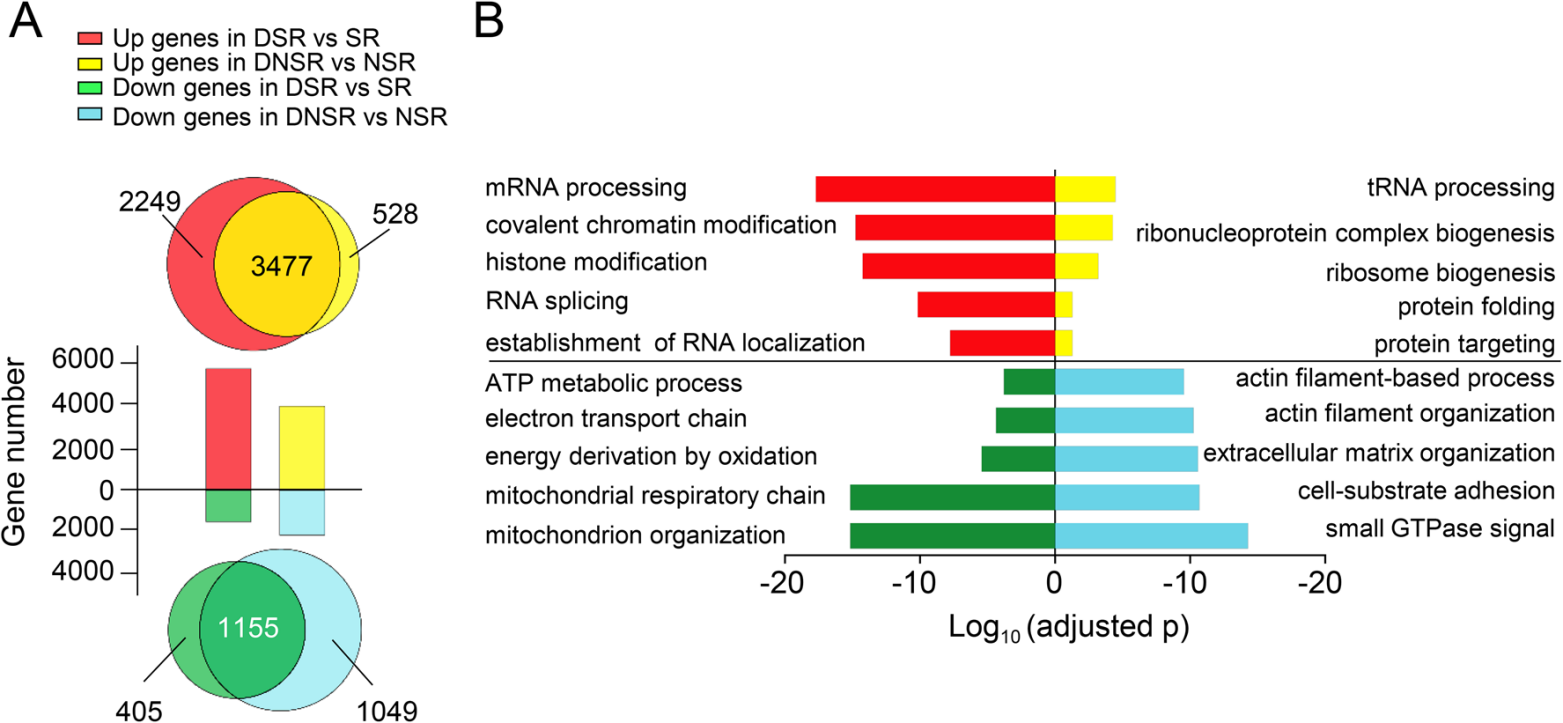
Different response to denervation in synaptic and non-synaptic region. **A** Venn diagram of DEGs in the synaptic region after denervation versus that in the non-synaptic region. The upper row showing the genes that were up regulated after denervation. The lower row showing the genes that were down regulated after denervation. **B** GO functional enrichment analysis of DEGs that were specifically regulated by denervation in synaptic and non-synaptic region shown in **A**

After denervation, we also noticed that some DEG_SR-DSR_ and DEG_NSR-DNSR_ changed with the same tendency, suggesting that these DEGs were co-regulated regardless of regional effect in myofibers. We removed those genes that were co-regulated upon denervation for subsequent analysis (Fig. 5A). After denervation, 5726 genes were up-regulated and 1560 genes were down-regulated in SR; while 4005 genes were up-regulated and 2204 genes were down-regulated in NSR. 2249 genes were specifically up-regulated in SR, and 528 genes were specifically up-regulated in NSR; 405 and 1049 genes were specifically down-regulated in SR and NSR respectively (Fig. 5A). Strikingly, those specific increased genes in DSR are mostly related to chromatin modification, while the decreased genes in the same region linked to energy metabolism (Fig. 5B). The function related to protein synthesis is enriched by the genes with high expression in DNSR, and on the other hand, the down-regulated genes in this region were mainly associated with cytoskeleton construction. Together, there is different response to denervation in SR and NSR. The regulation of chromatin modification seems to play an important role in the denervated synaptic region.

### Weighted correlation network analysis (WGCNA) revealed Kdm1a as a negative regulator of NMJ gene expression

**Fig. 6:**
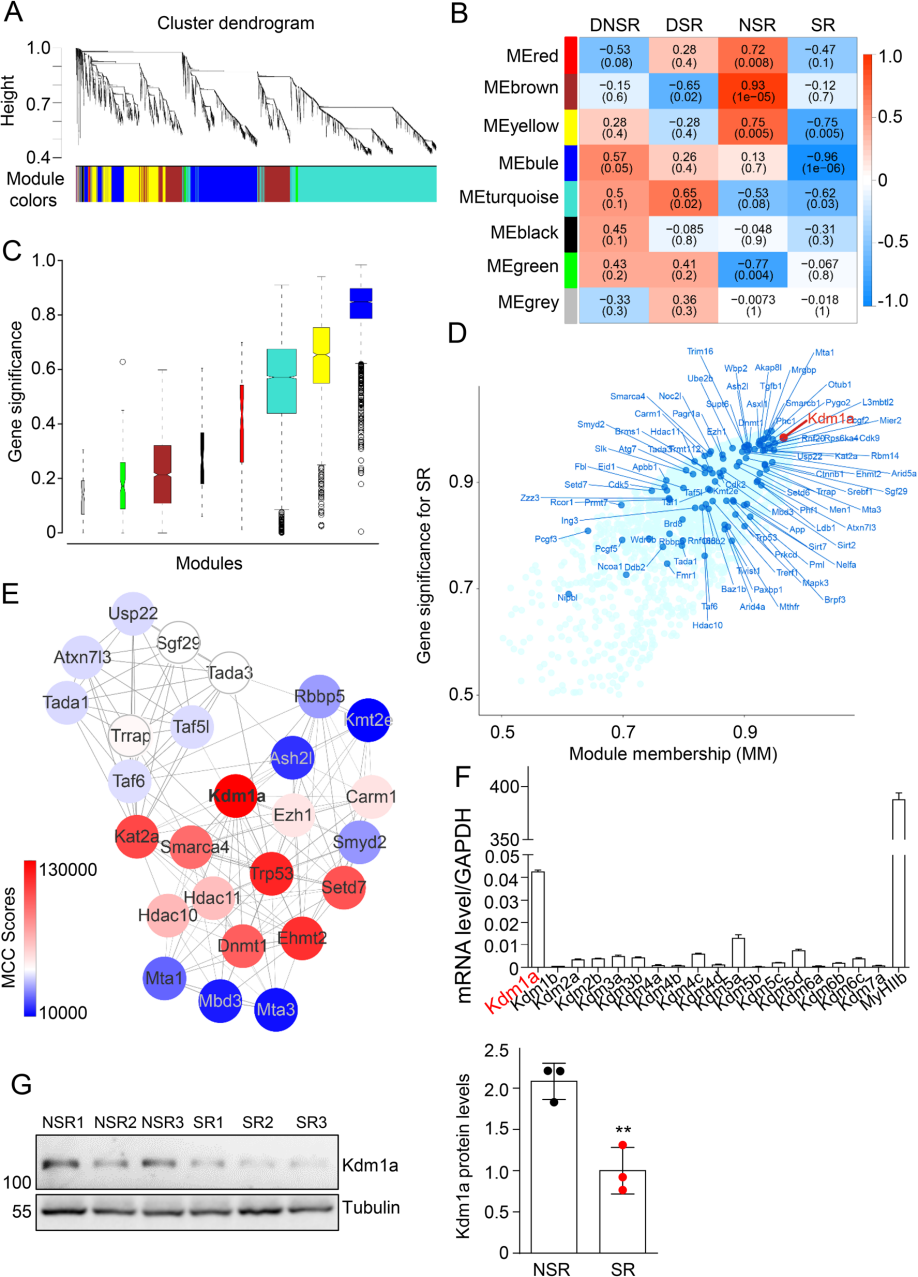
WGCNA revealed Kdm1a as a negative regulator of NMJ gene expression. **A** A total of 11,000 genes expressed in NSR and SR with or without motoneuron innervation were analyzed by WGCNA across 12 samples. According to their expression patterns, genes were clustered as shown by the dendrogram. Clusters of genes showing similar expression profiles are identified as modules and marked by different colors. It could be assigned into eight modules. **B** Heat-map showed Pearson correlation coefficients and significance levels between modules and different muscle conditions include NSR, SR, DNSR, and DSR. **C** Box plot showed the gene significance associated with SR in every module. **D** Genes assigned to the blue module were shown in the scatter plot. Genes related to histone modification were highlighted in navy blue. Module-Membership and gene significance of Kdm1a are 0.98 and 0.96 respectively. **E** STRING analysis of protein–protein interactions (PPI) from 93 genes related to histone modification in the blue module. The maximal clique centrality (MCC) was used to evaluate the interactome and only genes with MCC score over 10,000 were shown. **F** Real-time PCR analysis showing the highest expression of Kdm1a among lysine demethylase family members. TA muscles from 3-month-old mice were homogenized and mRNA were extracted for real-time PCR analysis. GAPDH was used as an internal control. MyHIIb was used as a control of muscles. **G** Immunoblot showing that the higher Kdm1a in NSR than those in SR of diaphragm muscles. n = 3 mice. Right, statics results. Unless otherwise specified, three independent experiments were performed; the mean ± SEM is shown. *t-test* in **p < 0.01

We have shown that the gene expression profiles of synaptic and non-synaptic regions assimilated upon the denervation with emphasis on the involvement of genes related to chromatin modelling. To comprehensively unravel the epigenetic regulator associated with a particular muscle condition, we analyzed 8 differentially regulated modules defined using WGCNA among 4 conditions (Fig. 6A) [18, 19]. In these 8 modules, the module that was highly correlated with SR is of great interest. Because it may play an important role in regulating NMJ genes not only when the nerve damage takes place but also under healthy conditions. The eigengene of the blue module showed a high negative correlation (-0.96) with the SR (Fig. 6B). In addition, the average of gene significance (the correlation of individual gene expressions and phenotypes) in the blue module was the highest, further supporting that the blue module is highly relevant to the SR (Fig. 6C). Next, we seek out the hub genes in the blue module in regard to the epigenetic change. The histone lysine demethylases Kdm1a showed the highest intra-modular connectivity and gene significance among 93 genes involved in histone modification (Fig. 6D), suggesting a hub role of Kdm1a in the module that was negatively correlated with the SR [32]. Next, we built the protein–protein interaction network (PPI) of the 93 genes in the blue module using the publicly available database STRING and analyzed the network using cytoHubba-plugin [33] (Fig. 6E). The topological analysis also indicated Kdm1a as the top hub port in the network (Fig. 6E). Real-time PCR analysis showed that Kdm1a expresses the highest among the family of histone lysine demethylases in TA muscles (Fig. 6F). The protein level of Kdm1a was higher in NSR compared to SR (Fig. 6G). Together, these data suggested that Kdm1a might be a key negative regulator of NMJ gene expressions.

### Kdm1a regulates AChR gene expression and NMJ functions.

**Fig. 7:**
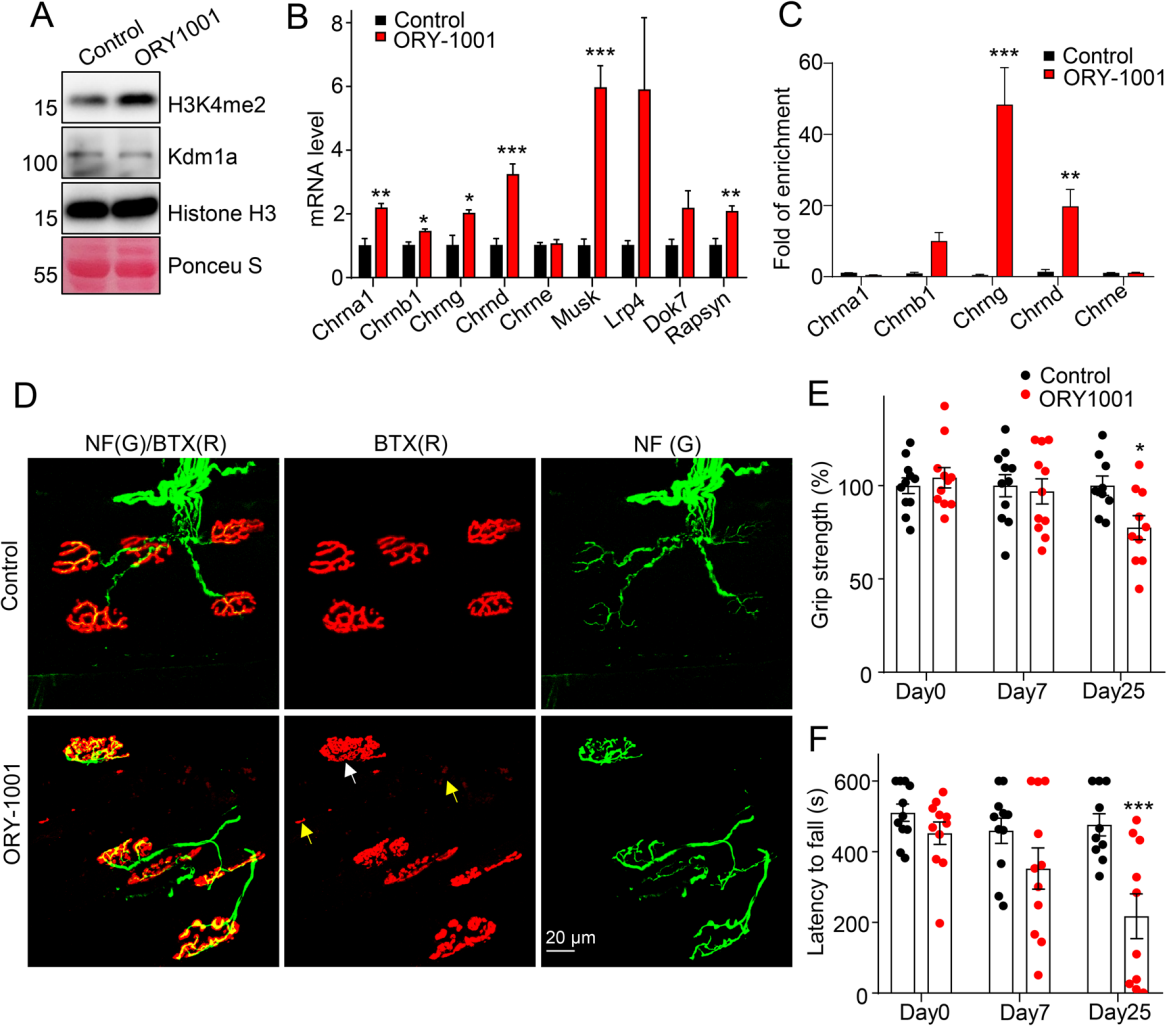
Kdm1a regulates AChR gene expression and NMJ functions. **A** Immunoblot showing the enhancement of H3K4me2 after ORY-1001 injection in TA muscles. **B** Real-time PCR analysis of synaptic gene expression in TA muscles with the ORY1001 treatment. Three mice per group. *t-test*. **C** ChIP assay showing ORY-1001 treatment promotes H3K4me2 to bind with the promoters of AChR subunits. **D** Whole-mount staining of TA muscles in mice after ORY-1001 injection (Day25). R-BTX (Red); NF (Green). **E** Mouse grip strength at indicated days after vehicle or ORY-1001 treatment. 11 mice in each group on day 0 and 7. n = 9 mice in control group and n = 10 mice in ORY-1001 group on day 25. Two-way ANOVA with Sidak’s post hoc test for multiple comparisons. Day: F (2,57) = 2.766; treatment F (1,57) = 2.271. **F** Rotarod analysis at indicated days after vehicle or ORY-1001 treatment. 11 mice in each group on day 0 and 7. 10 mice in each group on day 25. Two-way ANOVA with Sidak’s post hoc test for multiple comparisons. Day: F (2,58) = 4.874; treatment F (1,58) = 16.2. Unless otherwise specified, three independent experiments were performed; the mean ± SEM is shown. *p < 0.05, **p < 0.01, and ***p < 0.001

We investigated if the manipulation of Kdm1a activity would alter the gene expression levels of synaptic genes. ORY-1001 is a highly potent and selective Kdm1a inhibitor that induces H3K4me2 accumulation on Kdm1a target genes [34]. TA muscles in wild-type mice were injected with ORY-1001. Indeed, H3K4me2 levels increased in ORY-1001-injected muscle lysates (Fig. 7A), indicating a successful inhibition of Kdm1a activity. ORY-1001 up-regulated expression of NMJ genes in denervated muscles (Fig. 7B). ChIP assay found that ORY-1001 treatment promoted H3K4me2 to bind with the promoters of AChR subunit genes in C2C12 myotubes (Fig. 7C).

We examined NMJ morphology after ORY-1001 injection (Day25). In control, NMJ exhibits prizel-like structure; however, NMJs are denser in ORY-1001-treated muscles. There were also spared AChR puncta. These results suggested that ORY-1001 increased AChR clusters at SR and NSR regions (Fig. 7D). We further performed motor behavior to test whether ORY-1001 promotes NMJ functions. Interestingly, both grip strength and rotarod performance in ORY-1001-injected mice were impaired (Fig. 7E and F), suggesting that extra AChR clusters interfered with normal neurotransmission at endplates, consistent with the idea that the proper motor functions must be precisely controlled by one NMJ per adult myotube. Together, our data suggest that Kdm1a is critical for NMJ gene expression and motor functions.

## Discussion

NMJ is a tiny structure connecting motoneurons and muscle fibers. It locates in the middle of myofibers and controls movement. Agrin signaling plays critical roles for AChR protein clustering and postsynpase assembly. The regulation occurs at the protein levels. Here, we revealed motoneuron innervation determines the distinct features of synaptic nuclei, which may largely depend on epigenetic regulation.

Skeletal myofibers contain multiple cell nuclei, which locates along the mature myotube periphery. Synaptic nuclei are located just adjacent to the postsynaptic apparatus and is known to express NMJ genes. We found that these synaptic nuclei are gradually accumulated during development, especially around two weeks after birth, a time window when synapse elimination and maturation actively occurs. Distinct location and gene expression of myonuclei are established during NMJ maturation, which is consistent with a recent report that the number of DEGs between SR and NSR increases from early development (P0) to adulthood [12]. In vivo live imaging analysis revealed that no loss of myonuclei after weeks of denervation [35]. We found that denervation diminishes the expression differences between synaptic nuclei and non-synaptic nuclei with a global alternation in nucleus chromatin organization, favoring the idea that innervation is critical for controlling muscle gene expression. The gene expression seems to be changed earlier than the dispersion of nucleus anchoring and alternation of DAPI-stained heterochromatin because there is no observable change in synaptic nucleus enrichment and chromocenters after denervation for 3 days. The rationale behind gene expression mediated by innervation is complicated. It probably requires a coordinated interaction between epigenetic regulation, agrin signaling, neuromuscular activity, and nerve secreted factors [36]. Notice that skeletal muscle is composed of intrafusal and extrafusal fibers. The extrafusal fibers are innervated mainly by α-motoneurons, and intrafusal fibers are specialized sensory organs proprioceptors that are mainly innervated by both sensory and γ-motorneurons. Different from the enrichment of synaptic nucleus at the contact sites between α-motoneuron and extrafusal fibers, there was few muscle nucleus at the contact sites between γ-motoneuron and intrafusal fibers (data not shown). Instead, muscle nucleus in intrafusal fibers forms nucleus bag or nucleus chain, which makes the muscle nucleus in intrafusal fibers even more complicated [37]. Thus, our findings suggest that the synaptic nucleus in extrafusal fibers is regulated by α-motoneuron innervation.

The transcriptional factors such as MyoD and Myogenin is induced after denervation (data not shown), suggesting that they play critical roles in the response to denervation. However, the upstream regulatory mechanism remains unclear. Previous reports showed that epigenetic factors such as HDACs are involved in denervation-induced gene expression change [38–40], suggesting that nerve-controlled epigenetic regulation is important for muscle gene expression. Our data suggested that a broad change of gene expression in SR and NSR after denervation was accompanied by the alternation of chromatin structure. Such a broad change in expression could be contributed by both transcriptional factors and epigenetic factors. Interestingly, the responses in SR and NSR to denervation were different. WGCNA revealed the histone lysine demethylases Kdm1a as a key negative regulator of NMJ gene expression. Kdm1a is known to play critical roles in myotube differentiation and muscle regeneration [41, 42]. Here we provide a novel role of Kdm1a in negatively regulating NMJ gene expression.

In muscles, in addition to myofibers which account for the majority of cell populations, there are myogenic stem cells, adipocytes, endothelial cells, lymphoid cells, smooth muscle cells, tenocytes, and Schwann cells etc. [43]. Although the percentage of transcript products from these cells is much lower than those from myofibers, they are mixed in RNA-seq samples and might interfere with the detection of the low abundant transcripts from myofibers. Single-cell RNA-seq technology provides a powerful tool to delicately dissect the subpopulation in each cell type. However, the large size of myofibers limits the application of single-cell RNA-seq in muscle directly. The single-nucleus RNA-seq would largely solve this problem, especially detecting mRNA in the nucleus or nucleus-attached [44]. In addition, differences between synaptic and non-synaptic nucleus could be determined by mRNA transcripts, stability, size, transportation, and location [45]. Although we cannot exclude other possibilities involved in mRNA levels, the distinct change of DAPI-stained chromatin organization suggested that mRNA transcripts are critically involved in the process. Further experiments such as combining laser capture, muscle-specific cell nucleus isolation, and Hi-C technology, would largely expand our understanding of gene expression in myofibers.

## Conclusion

Our results demonstrate that motoneurons innervation determines the distinct gene expressions in multinucleated myofibers. It suggests that denervation-mediated dysregulation of muscle gene expression might be one of the reasons for muscle atrophy and weakness in patients with motoneuron degenerative disorders or disability of mobility. Targeting the muscle nucleus could be a new target for treating neuromuscular disorders.

## Supplementary Information

**Additional file 1: Figure S1.** Difference of nucleus between SR and NSR at NMJs. A. Whole-mount staining of diaphragm muscles from 2-month-old C57BL/6J mice. Note that several nuclei are enriched at R-BTX-labeled synaptic sites (indicated with dotted lines). Up, diaphragm muscles. Down, a single muscle fiber. B. Quantification of number of nucleus in the synaptic region (SR) or the non-synaptic region (NSR) in A. n =16 myofibers. Results were from three mice. C. Representative images of isolated SR and NSR from soleus muscles. Red, R-BTX. D. Immunoblot showing the expression of Tom20 and H3K4me2 in SR and NSR of diaphragm muscles. E. Immunoblot showing the expression of Tom20 and Kdm1a in SR and NSR of diaphragm muscles during development. F. ATP levels in SR and NSR of diaphragm muscles. Unless otherwise specified, three independent experiments were performed; the mean ± SEM is shown. t-test in B, and E, *p < 0.05 and ***p < 0.001.

**Additional file 2: Figure S2.** Difference of nucleus between synaptic and non-synaptic regions at NMJs. A. Low-power images of denervated muscle in Figure 3A. B. Quantification of peri-synaptic nucleus number in Figure 3A. n = 33 NMJs in Sham; n = 28 NMJs in D3; n = 31 NMJs in D7; n = 31 AChR cluster area in D16; n = 29 AChR cluster area in D30. One-way ANOVA with Tukey’s post hoc test for multiple comparisons F (4,147) = 13.03. C. Grip strength in SOD1G93A mice at indicated ages. Control mice: n = 4 in P60; n = 4 in P90; n = 4 in P120; n = 3 in P150; SOD1G93A mice: n = 4 in P60; n = 3 in P90; n = 4 in P120; n = 5 in P150. Two-way ANOVA with Sidak’s post hoc test for multiple comparisons. Age: F (3,23) = 9.158; genotype F (1,23) = 141.3. D. Low-power images of muscle in SOD1 mice in Figure 3E. E. DAPI staining in SR/NSR in TA mice from control and SOD1G93A mice at indicated ages. DAPI (blue), BTX (Red). Unless otherwise specified, three independent experiments were performed; the mean ± SEM is shown. *p < 0.05, **p < 0.01, and ***p < 0.001.

**Additional file 3: Figure S3.** Figure S3. Synaptic gene expression A. Real-time PCR analysis of representative synaptic gene expression in SR and NSR. Three repeats. T-test, mean ± SEM, *p < 0.05, **p < 0.01, and ***p < 0.001. B. Immunoblot showing the Kdm1a levels after denervation.

## Data Availability

The data of the current study are available from the corresponding author on reasonable request.
